# Multipath colourimetric assay for copper(II) ions utilizing MarR functionalized gold nanoparticles

**DOI:** 10.1038/srep41557

**Published:** 2017-02-03

**Authors:** Yulong Wang, Limin Wang, Zhenhe Su, Juanjuan Xue, Jinbo Dong, Cunzheng Zhang, Xiude Hua, Minghua Wang, Fengquan Liu

**Affiliations:** 1 Institute of Plant Protection, Jiangsu Academy of Agricultural Science, College of Plant Protection, Nanjing Agricultural University, Nanjing, P.R. China; 2 College of Plant Protection (Key Laboratory of Integrated Management of Crop Diseases and Pests), Nanjing Agricultural University, Nanjing, P. R. China

## Abstract

We use the multiple antibiotic resistance regulator (MarR), as a highly selective biorecognition elements in a multipath colourimetric sensing strategy for the fast detection of Cu^2+^ in water samples. The colourimetric assay is based on the aggregation of MarR-coated gold nanoparticles in the presence of Cu^2+^ ions, which induces a red-to-purple colour change of the solution. The colour variation in the gold nanoparticle aggregation process can be used for qualitative and quantitative detection of Cu^2+^ by the naked eye, and with UV–vis and smartphone-based approaches. The three analysis techniques used in the multipath colourimetric assay complement each other and provide greater flexibility for differing requirements and conditions, making the assay highly applicable for Cu^2+^ detection. Under optimal conditions, the Cu^2+^ concentration was quantified in less than 5 min with limits of detection for the naked eye, UV–vis and smartphone-based approaches of 1 μM, 405 nM and 61 nM, respectively. Moreover, the sensing system exhibited excellent selectivity and practical application for Cu^2+^ detection in real water samples. Thus, our strategy has great potential for application in on-site monitoring of Cu^2+^, and the unique response of MarR towards copper ions may provide a new approach to Cu^2+^ sensing.

To prevent contaminants from causing damage to human health and environmental catastrophes, it would be ideal to detect such contamination events as quickly as possible to be able to rapidly initiate remedial strategies^1^. Toxic metals such as Cu^2+^, a major environmental pollutant, have adverse effects on the self-purification ability of water systems in nature^2^,^3^. In addition, elevated concentrations of Cu^2+^ cause serious gastrointestinal disturbance^4^, liver and kidney damage^5^, and various neurological diseases^6^,^7^. The most commonly used detection methods for Cu^2+^ include inductively coupled plasma mass spectrometry^8^, atomic absorption spectroscopy^9^ and inductively coupled plasma optical emission spectroscopy^10^ (ICP-OES). Although these are very sensitive and quantitative techniques, they require either laborious sample preparation procedures or inconvenient analytical methods and are thus very time-consuming. Therefore, there is an ever-increasing demand for the development of quantitative techniques for the detection of Cu^2+^ that do not require advanced instruments.

In this context, colourimetric assays are very promising on account of their simplicity and cost effectiveness^11^. In particular, functionalised gold nanoparticles (AuNPs) have received considerable attention for use in colourimetric sensing because of their ease of biofunctionalisation, high extinction coefficients and characteristically strong surface plasmon resonance (SPR) absorption^12^,^13^,^14^. In most cases, AuNPs are used as chemical sensors by monitoring their colour changes on aggregation/dissociation because the colourimetric response can be easily observed with the naked eye. Recently, several AuNP-based colourimetric assays have been developed for Cu^2+^ sensing by modifying the AuNP surfaces with aptamers^15^, polyamines^16^, proteins^17^ and other specific reagents^18^. Zhou *et al*.^19^ reported the visual detection of Cu^2+^ using azide- and alkyne-functionalised AuNPs on the basis of a Cu^+^-catalysed alkyne–azide cycloaddition reaction. This method showed high specificity, but the minimum concentration of Cu^2+^ detectable by eye was approximately 50 μM. Accordingly, based on a similar Cu^+^-catalysed alkyne–azide cycloaddition reaction, Shen *et al*.^20^ further developed a simple “clickable” biosensor for colourimetric detection of Cu^2+^. This method had a low detection limit of 250 nM and a linear range of 0.5–10 μM. However, the click reaction is time-consuming and requires the alkyne/azide pair as well as the reduction of Cu^2+^ to Cu^+^, and thus limits the practical on-site application of the method. Wang *et al*.^21^ reported the visual detection of Cu^2+^ on the basis of Cu^2+^-dependent DNAzyme cleaving. Enzymatic cleavage techniques have shown considerable progress in terms of sensitivity, however, all such approaches require nuclease or protein enzymes, which make these methods inconvenient and not cost-effective. Guo *et al*.^17^ also demonstrated a colourimetric method for the detection of one or all of Hg^2+^, Pb^2+^ and Cu^2+^ using papain-functionalised gold nanoparticles. This method demonstrated simple, cost-effective and rapid performance but lacked specificity for Cu^2+^. Therefore, there is continuing interest in the search for new specific recognition elements that are highly selective for Cu^2+^ and show a quick response in environmental and biological samples.

The multiple antibiotic resistance regulator (MarR) proteins are an important family of regulatory proteins that regulate the transcription of a wide array of genes required for virulence and pathogenicity of bacteria^22^. These proteins are involved in various cellular processes, such as resistance to multiple antibiotics and adaptation to different environments^23^,^24^. Hao *et al*.^25^ have reported that Cu^2+^ potentiated MarR (a member of the MarR family from *Escherichia coli*) derepression by inducing dissociation of MarR from its cognate promoter DNA in *Escherichia coli*. Recently, our studies on the function of MarR in *Lysobacter enzymogenes* OH11 have shown that MarR from *Lysobacter enzymogenes* OH11 has an unexpected selectivity for Cu^2+^. Based on this result, we expected that the MarR could act as a novel specific recognition element for Cu^2+^; however, a literature survey revealed that no work has been published on Cu^2+^ detection using MarR.

In recent years, with advances in the computational capability of smartphones, smartphone-based colourimetric sensors have attracted increasing attention^26^,^27^,^28^,^29^,^30^. Colourimetric data obtained from digital images can be converted to analyte concentrations with a built-in smartphone app or Adobe Photoshop CS6 software by calculating the RGB values^28^,^29^. Smartphone-based colourimetric methods have the advantages of low cost, portability, possibility for on-site image recording and ease of implementation, and thus provide a new direction for colourimetric assays.

In this report, for the first time, we develop a multipath colourimetric assay for Cu^2+^ in water samples that uses MarR as the highly Cu^2+^ -specific biorecognition element. It was expected that Cu^2+^ would induce the aggregation of MarR-coated AuNPs (M–AuNPs) through tetramer formation of MarR, which is caused by Cu^2+^ oxidization. This aggregation caused the SPR absorption band of M–AuNPs to shift to longer wavelengths, and consequently a colour change from red to purple or grey was observed. Here, we use the naked eye, and UV–vis and smartphone-based approaches for the colourimetric analysis. The multipath nature of the assay meets the various requirements of different detection conditions, and the advantages of each approach complement those of the others. The advantages and disadvantages are highlighted in the following aspects: (i) direct detection with the naked eye does not require any instrumentation, but lacks quantitative analysis and generates uncertainty because of personal subjectivity; (ii) UV–vis analysis achieves quantitative detection with high accuracy, but always requires a professional UV–vis spectrometer; and (iii) the smartphone-based approach uses universal Adobe Photoshop CS6 software, resolves the requirement for professional instrumentation and provides quantitative analysis, which requires strict control of the light conditions for accurate test reading. In summary, our multipath colourimetric assay with the combined advantages of the three techniques provides greater flexibility and more options for a variety of requirements and conditions, and thus exhibits good applicability for the detection of Cu^2+^. In addition, the detection procedure can be performed at room temperature, and less than 5 min was required for the assay, which makes it a promising sensing platform for the rapid on-site detection of Cu^2+^. We thus believe that the unique response of MarR towards copper ions will provide a new approach to Cu^2+^ sensing.

## Methods

### Chemicals and instrumentation

Chloroauric acid (HAuCl_4_), trisodium citrate and copper sulfate were purchased from Sigma–Aldrich (St. Louis, MO, USA). Other metal ions salts (Na_2_SO_4_, KNO_3_, Ca(NO_3_)_2_, Cd(NO_3_)_2_, Mg(NO_3_)_2_, Pb(NO_3_)_2_, Zn(NO_3_)_2_, HgSO_4_, Mn(NO_3_)_2_ and Fe(NO_3_)_3_) and environmental anion salts (KCl, KNO_3_ and K_2_SO_4_) were obtained from Aladdin Reagent Co. Ltd. (Shanghai, China). All chemical reagents were of analytical grade and used without further purification. Ultrapure water with an electrical resistance greater than 18.2 MΩ was used for all the experiments. All glassware was thoroughly cleaned with freshly prepared 3:1 HCl/HNO_3_ and rinsed with ultrapure water before use.

UV–vis spectra were measured on a multifunctional microplate reader SpectraMax M5 (Molecular Devices, USA). Transmission electron microscope (TEM) images were obtained with a JEM-1200EX microscope (JEOL, Japan). The captured images were recorded with a smartphone (Huawei, Ascend P7, China) with back camera (13 Megapixel: 4160 × 3120 pixels) and the RGB values of the colourimetric detection results were acquired using Adobe Photoshop CS6 software.

### Preparation of MarR

Wild-type MarR and its mutant (MarR^Ser^) with C-terminal His-tags were expressed in *E. coli* BL21(DE3), which bears a pET30a vector containing the *marR* gene from *Lysobacter enzymogenes* OH11. Cells were grown in LB medium containing 30 μg mL^−1^ kanamycin at 37 °C to an OD_600_ of 0.6, and then isopropyl β-D-1-thiogalactopyranoside was added to a final concentration of 1 mM. Protein expression was induced at 18 °C for 12 h before harvesting. The cell pellets were collected and resuspended in protein extract buffer (50 mM Tris-HCl, pH 7.5, 100 mM NaCl and 1 mM ethylenediaminetetraacetic acid (EDTA)) containing 1 mM phenylmethanesulfonyl fluoride. After sonication on ice for 20 min, the cell lysate was centrifuged at 12,000 rpm for 10 min. The cleared lysate was filtered and loaded onto a 1-mL Histrap column (GE Healthcare), which was pre-equilibrated with 10 mL binding buffer (50 mM Na_3_PO_4_, 30 mM NaCl and 10 mM imidazole). The MarR protein was then eluted with a linear imidazole gradient followed by a further purification step using Amicon Ultra (Millipore). The protein was collected and concentrated in phosphate-buffered saline (PBS, 0.15 M, 1.47 mM KH_2_PO_4_, 8.09 mM Na_2_HPO_4_·12H_2_O, 136.9 mM NaCl, 2.68 mM KCl, pH 7.4) for further experimental use.

### Synthesis of AuNPs

AuNPs with an average diameter of 20 nm were prepared by a previously reported citrate-mediated reduction of HAuCl_4_^31^ with slight modifications. Briefly, a HAuCl_4_ solution (0.01%, l00 mL) was brought to boil in a 250 mL conical beaker with vigorous stirring, and then trisodium citrate solution (1%, 1.8 mL) was added under constant stirring. The colour of the solution changed to wine-red after about 45 s, The solution was boiled for a further 5 min, then the heating source was removed and the colloidal gold solution was stirred for another 10 min at room temperature. The solution was stored in a dark bottle at 4 °C. The concentration of the AuNP colloid was estimated to be about 9.7 nM from UV–vis spectroscopic measurements according to the Beer–Lambert law and based on an extinction coefficient of 8.78 × 10^8^ M^−1^ cm^−1^ at λ = 520 nm for 20 nm particles^32^.

### Preparation of MarR-coated gold nanoparticles (M–AuNPs)

To estimate the minimum quantity of MarR required to prevent the aggregation of colloidal gold, a salt-induced AuNP aggregation test was performed. In brief, the pH of the AuNP solution (20 nm mean diameter) was adjusted to pH 8.2 with K_2_CO_3_ (0.2 mol L^−1^), and serially diluted MarR solutions (30 μL, 62.5–1000 mg L^−1^) were added to portions of the AuNP solution (125 μL). After 5 minutes, NaCl solution (10%, 125 μL) was added to the mixture. The absorbance at 520 nm was measured before and after NaCl addition. More than 10% of minimum amount of MarR (0.22 mg) was added to the colloidal gold solutions (6 mL, pH 8.2) with rapid stirring, and this was followed by incubation for 4 h. The M–AuNPs were centrifuged three times (10,000 × *g*) at 4 °C for 25 min. After removal of the supernatant, the sediment was resuspended in ultrapure water at a concentration of 3.2 nM.

### Assay procedure for the colourimetric determination of Cu^2+^

In a typical experiment, a Cu^2+^ sample solution was added to a solution of M–AuNPs (120 μL), to give final Cu^2+^ concentrations in the range of 100 nM to 20 μM. After vibration for 2 min at room temperature, the colour changes were detected by the naked eye, UV−vis absorption spectroscopy and the smartphone-based approach. For detection with the naked eye, photographs were taken with a digital camera. The UV−vis analysis used absorption measurements obtained on a UV−vis spectrophotometer between 450 and 800 nm. For the smartphone-based approach, photographs were taken under artificial white light without flash using a smartphone equipped with a high-resolution camera. Image processing was performed by calculation of the RGB values by placing a 100 × 100 pixel box on each well using Adobe Photoshop CS6 software. The linear relationship was calculated from the A_600_/A_520_ absorbance ratio and the blue/red ratio versus the Cu^2+^ concentration, respectively.

### Analysis of real water samples

Tap water and commercial bottled water samples were analysed without filtration or any other pre-treatment. River water samples (Xuanwu Lake, Nanjing, China) were filtered through 0.45 μm cellulose acetate filters and the pH was adjusted to pH 7.0. Because the concentrations of Cu^2+^ in the water samples were lower than the detection limit of the proposed method, various amounts of Cu^2+^ standard solution were spiked into the water samples. These sample solutions were mixed with the pre-concentrated M–AuNPs to give a final concentration of 3.2 nM, then analysed with the multipath colourimetric assay.

## Results and Discussion

### Principle of the colourimetric assay

**Figure 1:**
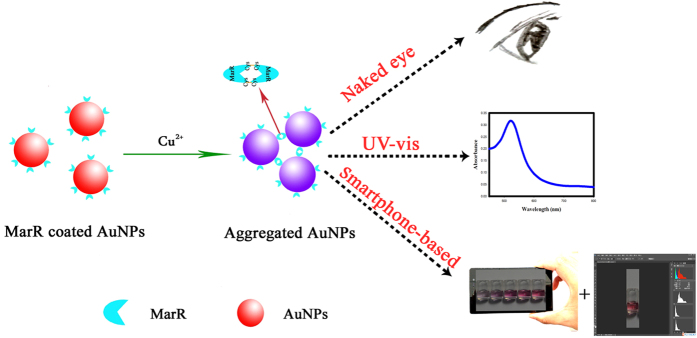
Schematic representation of the proposed Cu^2+^ sensing mechanism of the multipath colourimetric assay.

As shown in Fig. 1, MarR is a protein that is easily attached to the surface of AuNPs, and the M–AuNPs show good dispersion with a visible red colour. The single surface plasmon absorption peak is centred at 520 nm. In the presence of Cu^2+^, the M–AuNPs are aggregated and the colour changes from red to purple as the Cu^2+^ concentration is increased. As a result of the aggregation, the intensity of surface plasmon resonance band at around 520 nm decreases and a new peak appears at 600 nm^33^. The colourimetric images were also recorded using a smartphone and processed with Adobe Photoshop CS6 by calculation of the RGB values. Thus, the colour variation of the M–AuNP solution can be used for qualitative and quantitative detection of Cu^2+^ by the naked eye, UV–vis spectrophotometry and a smartphone-based approach.

**Figure 2:**
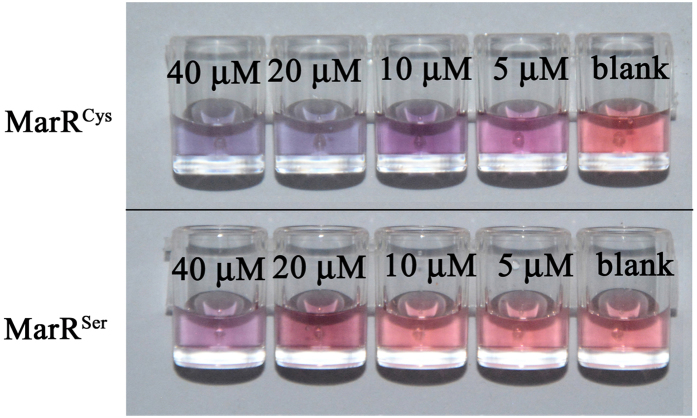
Colourimetric response of wild-type MarR (MarR^Cys^) and mutant (MarR^ser^) functionalized gold nanoparticles versus the Cu^2+^ concentration.

According to a previous study^25^, Cu^2+^ can oxidise the cysteine residue of MarR to generate disulfide bonds between two MarR dimers, thereby inducing tetramer formation. In our analytical system, MarR-coated AuNPs were used as the detection probe, and we postulated that the aggregation of M–AuNPs was induced by bridge formation between two M–AuNPs through catalytic oxidation of the cysteine residues promoted by Cu^2+^. To validate this assumption, we mutated the only cysteine residue of MarR to serine and used this mutant MarR in the Cu^2+^-induced colourimetric assay. As shown in Fig. 2, the wild-type MarR (MarR^cys^) probe showed a marked red-to-purple colour change with increasing of Cu^2+^ concentration, whereas the mutant (MarR^ser^) probe showed a colour change only when the Cu^2+^ concentration exceeded 20 μM. This indicates that the MarR^cys^ probe exhibits a more sensitive response to Cu^2+^ than does the MarR^ser^ probe. Therefore, we deduced that the aggregation mechanism in the colourimetric assay is based on the catalytic ability of Cu^2+^ to oxidise the cysteine residue of MarR.

### Characterization of M-AuNPs

**Figure 3:**
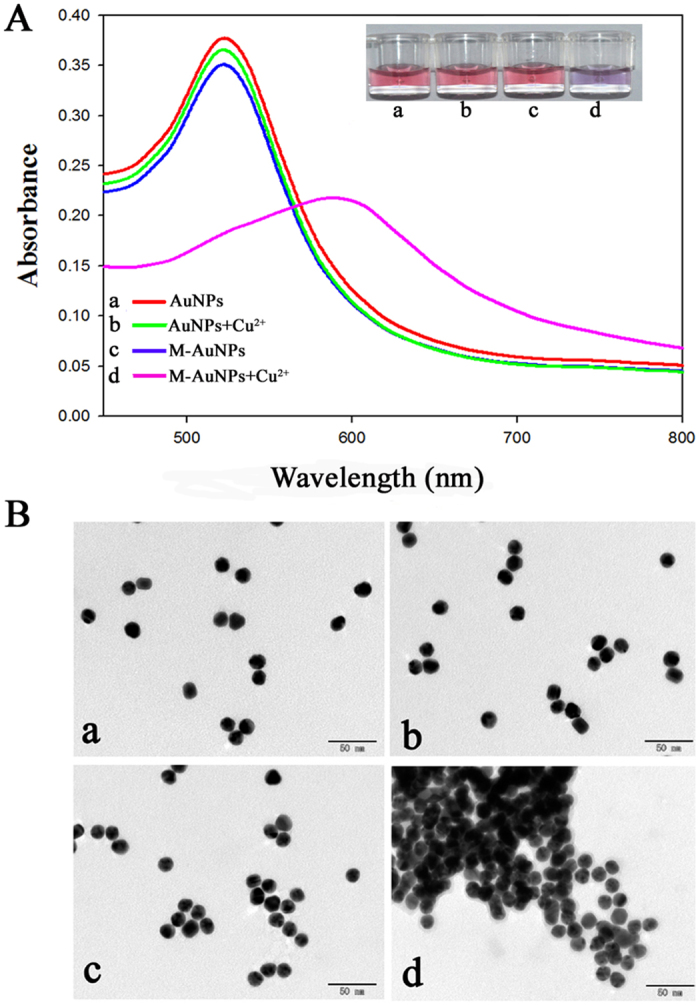
(**A**) Optical absorption spectra of gold nanoparticle (AuNP) solutions: (a) bare AuNPs; (b) multiple antibiotic resistance regulator (MarR)-coated AuNPs (M–AuNPs); (c) AuNPs+Cu^2+^; (d) M–AuNPs+Cu^2+^. Inset of (**A**): photographic image of the AuNP dispersion showing that the colour change occurs only in the presence of M–AuNPs and Cu^2+^ (20 μM). (**B**) Corresponding transmission electron microscopy images (scale bars: 50 nm).

To investigate the viability of our strategy, the M–AuNPs were characterised in the presence and absence of Cu^2+^ by UV–vis spectroscopy and TEM. As shown in Fig. 3A, the characteristic band of the AuNPs and M–AuNPs was observed at approximately 520 nm (curves a, c), and the colour of the solution appeared red (photographs a, c). After addition of an appropriate amount of Cu^2+^, the colour of the M–AuNP solution changed from red to purple (photograph d), and this colour change was confirmed by the dramatic decrease in the absorbance at 520 nm and the appearance of an intense new peak at about 600 nm (curve d). In contrast, the colour of the AuNP solution remained red after Cu^2+^ treatment (photograph b), indicating that the aggregation of AuNPs was mainly caused by the interaction between MarR and Cu^2+^. TEM studies (Fig. 3B) clearly indicated the Cu^2+^-induced aggregation of M–AuNPs, and thus confirmed the validity of our strategy.

### Optimisation of assay conditions

**Figure 4:**
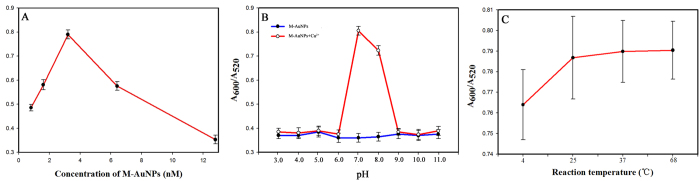
Effect of the concentration of the M–AuNPs (**A**), the pH value (**B**) and the reaction temperature (**C**) on the signal response of the colourimetric assay. The absorbance ratio value (A_600_/A_520_) were obtained by UV–vis spectrophotometry. Cu^2+^ concentration was 4 μM. Error bar at n = 3.

To optimise the performance of the colourimetric assay, it was necessary to consider the concentration of the M–AuNPs, the pH value and the reaction temperature. To investigate the influence of the M–AuNP concentration, the ratio of the absorbances at 600 and 520 nm (A_600_/A_520_) was used to reflect the signal response of the sensor. As shown in Fig. 4A, the maximum A_600_/A_520_ absorbance ratio was observed when the concentration of M–AuNPs was 3.2 nM. An M–AuNP concentration of 3.2 nM was thus chosen for further experiments to achieve highly sensitive Cu^2+^ detection,

Next, we investigated the colourimetric response of M–AuNPs with Cu^2+^ at different pH values (pH 3.0–11.0). The correlation of the A_600_/A_520_ absorbance ratio with and without Cu^2+^ at a range of pH values is shown in Fig. 4B. The absorbance ratio was low and constant in the absence of Cu^2+^ from pH 3.0 to 11.0, which indicates that the M-AuNPs were stable in this pH range. Addition of Cu^2+^ resulted in a high A_600_/A_520_ absorbance ratio at pH 7 and 8, which indicates that the highest catalytic efficiency of Cu^2+^ towards the cysteine residue of MarR occurs at these pH values. In our experiments, a higher absorbance ratio was observed at pH 7.0 than at pH 8.0, so pH 7.0 was considered to be the optimal pH value.

We also tested the effect of temperature, from 0 to 68 °C, on the sensor performance. As shown in Fig. 4C, the A_600_/A_520_ absorbance ratio changed only very slightly over the studied temperature range, suggesting that the catalytic efficiency of Cu^2+^ towards MarR was retained over a wide temperature range. For easy implementation and extensive application of our assay, we chose room temperature for the following experiments.

### Sensitivity of the Cu^2+^ sensor

**Figure 5:**
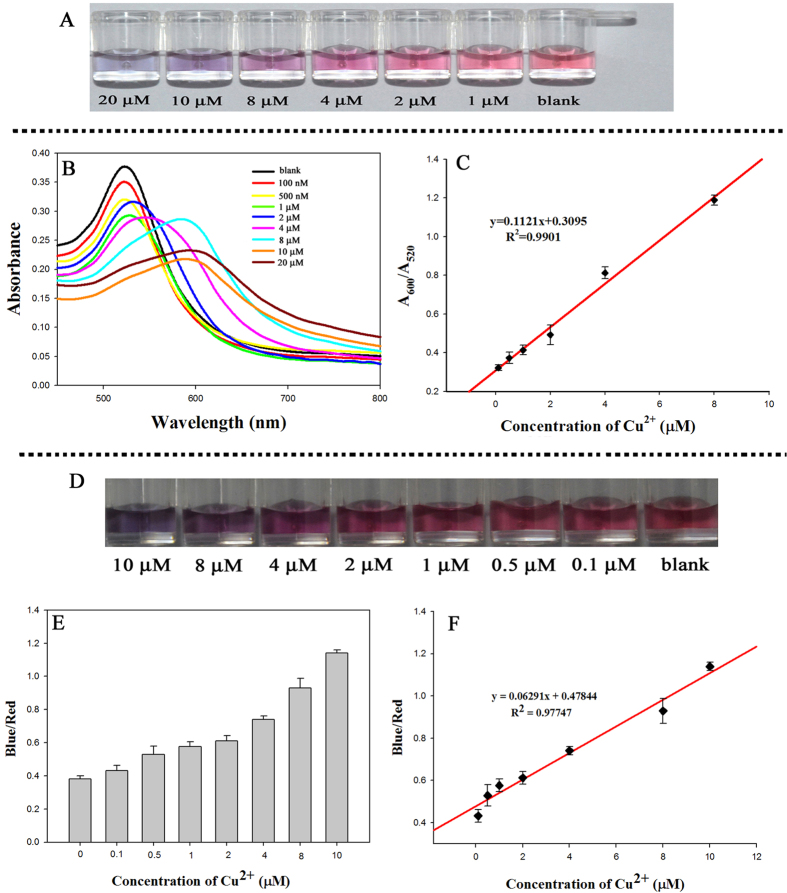
Colourimetric sensing of Cu^2+^. (**A**) Photographs of multiple antibiotic resistance regulator (MarR)-coated AuNP (M–AuNP) solutions with different concentrations of Cu^2+^ in the range from 100 nM to 20 μM. (**B**) UV–vis spectra of the M–AuNP solutions. (**C**) Plot of the A_600_/A_520_ absorbance ratios of M–AuNP solutions versus the concentrations of Cu^2+^. (**D**) Photographs of M–AuNP solutions with different concentrations of Cu^2+^ using a smartphone under artificial white light without flash. (**E**) and (**F**) Plot of blue/red colour values of the smartphone photographs versus the concentrations of Cu^2+^. Error bars at n = 3.

To determine the sensitivity of the multipath colourimetric assay, Cu^2+^ concentrations between 100 nM and 20 μM were tested under the optimised conditions. For naked-eye detection and UV–vis analysis, the A_600_/A_520_ absorbance ratio was recorded for each concentration of Cu^2+^, as well as the corresponding colourimetric response. As shown in Fig. 5A, with increasing concentrations of Cu^2+^, the colour of the M–AuNPs solution gradually changed from red to purple and finally to grey, which suggests an increase in the aggregation of the M–AuNPs at higher Cu^2+^ concentrations. The intensity of the maximum absorption at 520 nm decreased and that of the new peak at 600 nm increased with increasing Cu^2+^ concentration (Fig. 5B), which also indicates the gradually increasing aggregation of the particles. Plots of the A_600_/A_520_ ratio versus the concentration of added Cu^2+^ exhibit good linear relationships in the range 0.1–8 μM Cu^2+^ (Fig. 5C), and the limit of detection (LOD) for Cu^2+^ was calculated to be 405 nM based on the following formula: LOD = average response of the blank + (3 × standard deviation of the blank)^11^. In the smartphone-based approach, the blue/red ratio obtained from the RGB values of each image (Fig. 5D) was calculated based on the colour change of the solution, and correlated with the increase in the concentration of Cu^2+^. The graphs in Fig. 5E and F show that the blue/red ratio increased as the concentration of Cu^2+^ increased, and a linear relationship was observed in the range 0.1–10 μM. The LOD was estimated to be 61 nM using the smartphone-based approach. These results compare favourably with other reported AuNP-based colourimetric methods (Table 1), and thus suggest that our MarR-functionalised AuNP-based colourimetric assay is superior to most previously reported methods in terms of speed, selectivity, sensitivity and number of analytical approaches.

### Selectivity of the Cu^2+^ sensor

**Figure 6:**
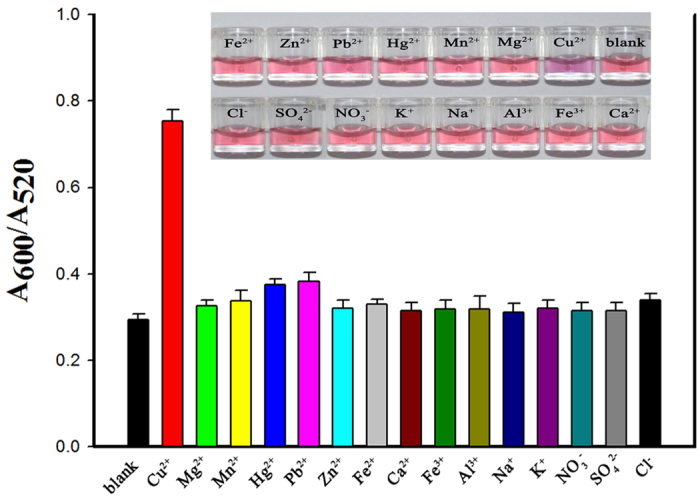
A_600_/A_520_ absorbance ratios of multiple antibiotic resistance regulator (MarR)-coated AuNPs (M–AuNPs) on addition of Cu^2+^ (4 μM), other metallic ions (10 μM; Mg^2+^, Mn^2+^, Hg^2+^, Pb^2+^, Zn^2+^, Fe^2+^, Ca^2+^, Fe^3+^, Al^3+^, Na^+^, K^+^) or environmental anions (1 mM; NO_3_^−^, SO_4_^2−^, Cl^−^). Error bars at n = 3. The inset shows the corresponding photographic images of the M–AuNP solutions.

To evaluate the selectivity of the colourimetric assay towards Cu^2+^, the effects of potential interfering metal ions Mg^2+^, Mn^2+^, Hg^2+^, Pb^2+^, Zn^2+^, Fe^2+^, Ca^2+^, Fe^3+^, Al^3+^, Na^+^ and K^+^ (10 μM), and environmental anions, Cl^−^, NO_3_^−^ and SO_4_^2−^ (1 mM) on the detection were investigated by UV–vis analysis. The concentration of Cu^2+^ was 4 μM. As shown in Fig. 6, addition of Cu^2+^ caused a colour change and the solution became purple, whereas none of the other metal ions markedly changed the colour of the M–AuNP solutions. The corresponding absorbance data also showed that only Cu^2+^ addition yielded a large increase in the A_600_/A_520_ ratio. It is worth noting that Pb^2+^ and Hg^2+^ caused a slightly larger colourimetric response than did the other metal ions. This may be attributed to their stronger coordination to cysteine residues and other functional groups (e.g., carboxyl, amino, hydroxyl) of the MarR protein^17^,^34^. However, the interaction between these groups and Pb^2+^ and Hg^2+^ is expected to be much weaker than the interaction between MarR and Cu^2+^ under the present conditions. The observation of a high absorbance ratio for Cu^2+^ demonstrates the excellent selectivity of our proposed method for Cu^2+^ over other competing metal ions.

### Application to real water samples

**Figure 7:**
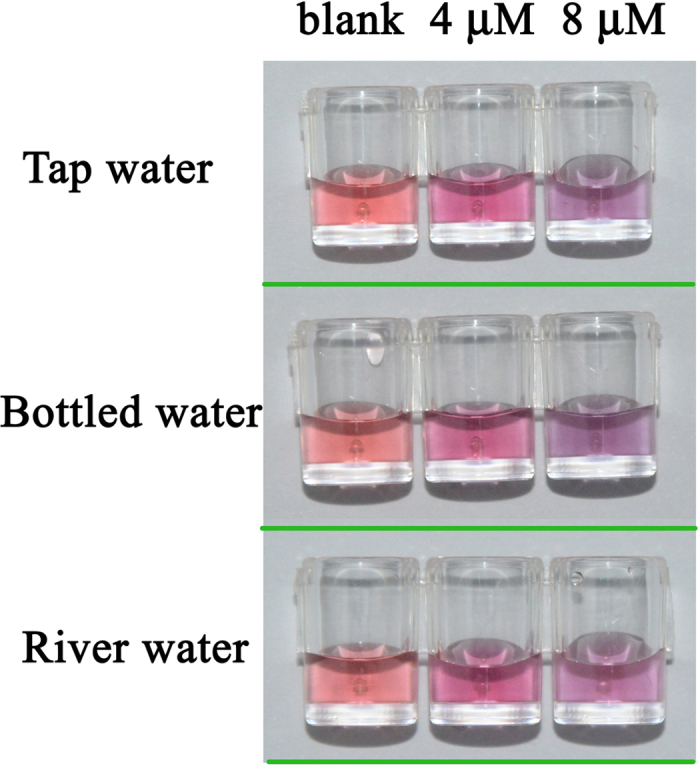
Sensing results for the naked eye detection of Cu^2+^ in tap water, bottled water and river water samples.

To illustrate the practical application of the multipath colourimetric assay, three water samples (tap water, river water and commercial bottled water) were spiked with different concentrations of Cu^2+^ and tested using the developed assay. The obtained results for the three analytical approaches are shown in Fig. 7 and Table 2. The results obtained with the present colourimetric method were in excellent agreement with those obtained by ICP-OES, and the spike-recoveries were 90.25–110.50%. These results demonstrate the potential of the developed multipath colourimetric assay for the visual detection of Cu^2+^ in real water samples.

## Conclusions

Using MarR as highly selective recognition element for Cu^2+^ detection based on the Cu^2+^-induced tetramer formation of MarR, we have developed a straightforward, rapid, sensitive and highly selective multipath colourimetric assay for Cu^2+^ in water samples. The Cu^2+^ assay can be easily implemented using the naked eye, UV–vis spectroscopy and a smartphone-based approach, which endows the colourimetric assay with great flexibility and applicability under an extensive range of conditions. The detection limit of the colourimetric assay is as low as 61 nM, which is far below the maximum recommended limit in drinkable water (20 μM) defined by the U.S. Environmental Protection Agency. Moreover, the whole test can be completed within 5 min. We envisage that this new Cu^2+^-regulated specific recognition element (MarR) may provide a simple and general approach to the selective detection of Cu^2+^, as well as a promising new tool for Cu^2+^ analysis based on the developed multipath colourimetric assay.

## Additional Information

**How to cite this article**: Wang, Y. *et al*. Multipath colourimetric assay for copper(II) ions utilizing MarR functionalized gold nanoparticles. *Sci. Rep.* **7**, 41557; doi: 10.1038/srep41557 (2017).

**Publisher's note:** Springer Nature remains neutral with regard to jurisdictional claims in published maps and institutional affiliations.

## Figures and Tables

**Table 1:** Comparison of different AuNPs-based colourimetric methods for the detection of Cu^2+^.

Probe unit	Linear range	LOD^b^	Time (min)	Interference ions	Analytical approach	Ref.
Papain–AuNPs	N/A^a^	200 nM	N/A^a^	Hg^2+^, Pb^2+^	naked eye and UV-vis	17
DNA–AuNPs	20–100 μM	20 μM	120	None	naked eye and UV-vis	18
Azide and alkyne–AuNPs	0.5–10 μM	250 nM	20–60	None	naked eye and UV-vis	20
DNAzyme–AuNPs	N/A^a^	5 μM	90	Zn^2+^	naked eye and UV-vis	35
Thermally treated AuNPs	0.2–6.0 μM	40 nM	5	Hg^2+^, Pb^2+^	naked eye and UV-vis	36
Azide-tagged AuNPs	1.8–200 μM	1.8 μM	30	Cr^3+^, Pb^2+^, Cd^2+^	naked eye and UV-vis	37
MarR–AuNPs	0.1–10 μM	61 nM	<5	None	naked eye, UV-vis and smartphone-based	This work

^a^Not available.

^b^limit of detection.

**Table 2:** Recovery studies of real water samples spiked with various Cu^2+^ concentrations, comparing the proposed colourimetric method with ICP-OES analyses.

Sample	Cu^2+^ (μM)	The proposed method						ICP-OES		
		UV-vis			Smartphone-based approach					
		Measured ± SD^a^ (μM)	Recovery^b^ (%)	CV^c^ (%)	Measured ± SD^a^ (μM)	Recovery^b^ (%)	CV^c^ (%)	Measured ± SD^a^ (μM)	Recovery^b^ (%)	CV^c^ (%)
Tap water	4	3.66 ± 0.19	91.50	5.19	3.81 ± 0.65	95.25	17.0	4.02 ± 0.24	100.50	5.97
	8	8.27 ± 0.54	103.38	6.53	8.02 ± 0.49	100.25	6.11	8.65 ± 0.63	108.13	7.30
Bottled water	4	4.26 ± 0.24	106.50	5.63	4.42 ± 0.25	110.50	5.66	4.13 ± 0.16	103.25	3.87
	8	8.06 ± 0.41	100.75	5.09	8.21 ± 0.28	102.63	3.41	8.35 ± 0.53	104.37	6.35
River water	4	3.95 ± 0.15	98.75	3.80	3.61 ± 0.35	90.25	9.70	4.30 ± 0.17	107.50	3.95
	8	7.41 ± 0.39	92.62	5.26	7.71 ± 0.50	96.38	6.50	8.19 ± 0.39	102.37	4.76

^a^Mean measured concentration of three replicates ± standard deviation.

^b^Mean recovery (%) = 100 × (c_mean measured_/c_added_).

^c^Coefficient of Variation of three determinations.
